# Tools for early screening of autism spectrum disorders in primary health care – a scoping review

**DOI:** 10.1186/s12875-022-01645-7

**Published:** 2022-03-15

**Authors:** Mateusz Sobieski, Aleksandra Sobieska, Małgorzata Sekułowicz, Maria Magdalena Bujnowska-Fedak

**Affiliations:** 1 Department of Family Medicine, Wroclaw Medical University, Wroclaw, Poland; 2 Department of Clinical Psychology and Health, SWPS University of Social Sciences and Humanities, Wroclaw, Poland; 3 Department of Social Sciences, University School of Physical Education, Wroclaw, Poland

**Keywords:** Autism spectrum disorder, Diagnostic screening programs, Primary health care

## Abstract

**Background:**

Autism spectrum disorder (ASD) is a neurodevelopmental disorder that manifests itself in early childhood. Early diagnosis of these disorders allows for the initiation of early therapy, which is crucial for the child's further functioning in society.

**Objectives:**

This review aims to gather and present the existing ASD screening tools that can be used in primary care and adapted to different countries conditions linguistically and culturally.

**Eligibility criteria:**

We searched for English-language publications on ASD screening tools for children aged 0–3 years suitable for use in primary care (i.e. free, requiring no additional training or qualifications).

**Sources of evidence:**

Four databases were explored to find English studies on ASD screening tools intended for the rapid assessment of children aged 0–3.

**Charting methods:**

The information sought (specific features of the questionnaires relevant to primary health care workers, psychometric and diagnostic values of a given cultural adaptation of screening tools, and the linguistic and cultural changes made) were extracted and collected to create profiles of these tools.

**Results:**

We found 81 studies which met inclusion criteria and underwent full data extraction. Three additional data sources were included. These allowed to create 75 profiles of adaptations for 26 different screening tools and collect data on their psychometric values and characteristic features.

**Conclusions:**

The results of our study indicate the availability of several diagnostic tools for early ASD screening in primary care setting concordant culturally and linguistically with a given population. They could be an effective method of accelerating the diagnostic process and starting personalized therapy faster. However, most tools have significant limitations – some are only available for research purposes, while others do not have scientific evidence to prove their effectiveness.

**Supplementary Information:**

The online version contains supplementary material available at 10.1186/s12875-022-01645-7.

## Introduction

Autism spectrum disorder (ASD) is a category of neurodevelopmental disorders characterized by challenges concerning social skills, speech development and behavior [1]. The cause of ASD is not known—it was suggested that the etiology includes many factors, including genetic, infectious or metabolic ones [2]. These disorders occur in all racial, ethnic and socioeconomic groups [3]. The prevalence is yet to be clearly defined; however, the World Health Organization (WHO) estimates that ASD occurs in 1 in 160 children worldwide [4]. However, this estimate varies considerably depending on the research method and country. For example, in Israel, it is 4.8%; in Iceland – 3.13%; in the United States – 1.7%; in Qatar – 1.14%; in Iran – 0.06% [5–10]. Thus, the percentage of individuals with ASD in the population depends primarily on diagnosis methods. The growing number of registered cases of ASD in recent years probably results from a greater number of diagnosed adults and children than changes in the frequency of the autism spectrum phenotype in the population [11].

Diagnosis of ASD is a long-term and multi-stage process aimed at recognizing existing disorders and assessing a child’s functioning on many levels. It begins with observing the child by parents, guardians, or other people who have contact with the child. It is also necessary to exclude other diseases that may cause symptoms similar to ASD. For this reason, consultations with other specialists (e.g., audiologists, laryngologists, geneticists) are necessary. The final stage is the definitive diagnosis by a team of specialists (psychiatrists, psychologists, special educators, or speech therapists) [12].

### The role of family doctors and pediatricians in early diagnosis of ASD

Family doctors or pediatricians working in a primary care clinic most often observe a child during infancy and early childhood, especially as part of well-child care visits, qualifications for vaccinations, or visits due to common infectious diseases. This fact enables careful observation of the child’s development and behavior in the critical period for diagnosing ASD, which means that the general practitioner (GP) may be first to notice the behavioral signs of disorders [13–15].

A desirable situation from the patients’ point of view is the GP taking the role of a “gatekeeper,” i.e., a person who notices the first “red flags” in the child’s behavior, analyzes the concerns raised by parents, and decides about the need for further specialist consultations [16]. During the aforementioned visits, parents ask questions about the symptoms they notice and express concerns about their child’s development [13]. Unfortunately, there are still frequent situations when doctors marginalize, minimize, or ignore the concerns raised by parents [17]. This may be due to organizational reasons related to primary health care structure (e.g., limited consultation time, excessive workload) [16, 18]. Moreover, identifying some ASD-specific features (e.g., sensory disorders) requires – apart from experience in this matter – devoting more time to patients than is generally provided for a visit in primary care clinics [19]. Another problem that hinders early diagnosis in primary care is the insufficient knowledge of doctors about ASD. A study conducted in 2020 showed that only 23% of primary care physicians (PCPs) had sufficient knowledge about ASD, and the percentage of such doctors was higher in countries with higher income [20]. For example, in Pakistan, only 44% of GPs knew the concept of autism, and only 42% of them had any further knowledge about it [21]. The driving force to improve the knowledge and skills of PCPs in the field of ASD may be the growing public awareness of the issue. Unfortunately, the spread of the term “autism” in society produced mixed results. On the one hand, greater awareness of the problem allowed many families to get help and additional financial resources; on the other hand, it also led to an uncontrolled public debate and spread of unfavorable stereotypes and untruths about ASD and its etiology [22–24]. A better method of spreading knowledge about ASD is special training for doctors by experts [25].

### Possibilities of early detection of ASD

Identification of autism spectrum disorders is challenging in the early stages of life when changes in development are rapid and symptoms – often subtle [26]. However, early diagnosis is a necessary first step to implement effective therapy appropriate to the child’s needs at a critical time of development – the younger the child at the time of ASD diagnosis, the better therapy results [27–29].

In order to increase the effectiveness of PCPs in the early diagnosis of ASD, numerous screening questionnaires have been developed, which their proponents claim to be some of the most beneficial health policy innovations ever created for children with ASD [30]. On the other hand, ASD screening is criticized in terms of cost-effectiveness or time constraints and the low psychometric properties of tests, especially in very young children [31–33]. However, there is evidence suggesting that including screening tools in routine medical appointments may result in earlier and more accurate identification of children who need further help than relying solely on clinical impressions, which is particularly important when care providers are less experienced in diagnosing ASD [34, 35]. Since the effectiveness of detecting ASD using various questionnaires (understood as the percentage of true positive results) increases with age, very early diagnosis of the youngest children is one of the major therapeutic problems. For such patients in whom screening is associated with tests of low psychometric properties, developmental follow-up is essential later in life. A solution to these problems may be developing novel and better diagnostic methods that take into account both the age and gender of the child [36].

Since 2006, the American Academy of Pediatrics (AAP) has recommended routine diagnosis of ASD at 18 and 24 months of age during well-child care visits [37]. Children who receive a positive screening result should be sent for further ASD evaluation to an early intervention center and referred to an audiologist to rule out hearing impairment, as recommended by the AAP [38, 39]. Over 14 years, these activities significantly increased the prevalence of ASD and made primary care facilities the main places of early diagnosis of ASD [40]. Following these recommendations resulted in more than 50% of American children undergoing screening for autism spectrum disorder [41–43]. In addition, the increasing availability of screening significantly lowered the age of ASD diagnosis in the US, with diagnosis before the age of 4 made in 71% of children (2018) compared to 58% in 2014 [40, 44].

In turn, the recommendations of the US Preventive Services Task Force indicate the lack of sufficient evidence in favor or disadvantage of performing ASD screening in children, for whom no concerns of ASD have been raised by their parents or a clinician [45].

### Aim of the study

The main aim of this scoping review was to demonstrate available, culture-specific and language-adapted tools for the early screening of autism spectrum disorders in children from 0 to 3 years of age, that can be used by healthcare professionals working in primary care. We were interested in gaining better insight into their psychometric properties and cultural adaptations, which is particularly important due to the social diversity of cultures [46]. Our final goal is to identify the most relevant tools for screening for ASD in primary care.

The collected data can be used by primary care professionals to select the best tool for the early diagnosis of ASD in their daily practice to accelerate the therapeutic process and for specialists in this field to highlight existing gaps.

## Materials and methods

In this research we used the five-step approach described by Arksey and O’Malley to conduct a scoping review: 1) identifying the research question, 2) identifying relevant studies, 3) selecting the studies, 4) charting the data, 5) collating, summarizing and reporting the results [47]. The whole process was dynamic and iterative, with each step discussed with a group of investigators. The Arksey and O’Malley’s framework is the primary method of conducting a scoping review which synthesizes the knowledge from the previous literature and allows to adapt the data for the purposes of the study. As the ambiguity of concepts remains the main disadvantage of this approach, when designing the study we also relied on the recommendations that appeared later e.g. Preferred Reporting Items for Systematic Reviews and Meta-Analyses extension for scoping reviews (PRISMA-ScR) (Additional file 1) [48]. We do not have a published protocol for this study.

### Identifying the research question

Our scoping review focused on answering the question: What are the suitable ASD screening questionnaires available that can be used in primary health care, and what are their characteristics? By suitability we mean a free (available in the public domain or after contact with the authors), short screening questionnaire, completed by a parent or clinician, characterized by good psychometric values and requiring no additional training in order to use it.

### Identifying relevant studies

The primary search strategy was developed collaboratively by all authors. We conducted an online search using four different scientific databases containing articles concerning medical and psychological sciences (PubMed, EBSCO, Scopus, and Web of Science) to find publications related to the early diagnosis of children with ASD. We used Mendeley to collect and organize the references. The search began in March 2021 and included all publications written in English and released from January 1980 to May 2021.

The initial search results included a large number of studies related to developmental screening processes and provided guidance and recommendations for the use of screening tools (e.g. AAP guidelines) [37]. The results also included research describing the development and validation of tools, the adaptation of screening tools, and comparisons between individual instruments. Using previously published scientific research on ASD screening tools and our literature search, we compiled a list of tools used for this purpose [49, 50].

As names of screening tools were not mentioned in the title or keywords of many peer-reviewed papers, we also performed individual searches to identify them. Therefore, at each stage of the search for screening tools (step 1), an individual search (step 2) was performed using the name of each instrument indicated in the general search results. In addition, we adapted the search string to the thesaurus of three other databases. Finally, using a snowball approach, we added articles of the reference lists if they met the inclusion criteria mentioned below but were not listed in the initial search. The exact terms we used in the searches can be found in S1 File.

Unfortunately, not all the information sought by us was available in peer-reviewed scientific publications. Therefore, we collected information about screening instruments from several sources. For example, we checked test reviews and articles describing psychometric properties in peer-reviewed journals, manuals, technical papers, doctoral dissertations, and information from test publishers or distributors.

### Inclusion criteria

1. Studies on tools intended for diagnosing children from 0 to 3 years of age;
2. Research describing the use of the tool published in English (or at least an abstract providing the necessary information);
3. Research on the tool intended for screening or rapid assessment, not a formal diagnosis of ASD.

### Exclusion criteria

1. Studies on tools intended for formal diagnosis (for this reason, instruments such as the Autism Diagnostic Observation Schedule (ADOS) or Bayley Scales of Infant Development (BSID) were excluded from the study). We also excluded more complex tools beyond the competencies of family doctors, requiring additional training or completion of training authorizing to use them (e.g., Ages and Stages Questionnaire (ASQ), Social Responsiveness Scale (SRS-2), Achenbach System of Empirically Based Assessment (ASEBA), Parents’ Evaluation of Developmental Status (PEDS), or Autism Spectrum Rating Scales (ASRS));
2. Studies on tools intended for screening children older than three years. For this reason, the publication omits, for example, the Social Communication Questionnaire (SCQ), which, according to the authors of the tool, is intended for screening of children over four years of age;
3. Research on diagnostic tools used in screening for other developmental disorders.

Information on screening tools was not always readily available; therefore, the decision to include a particular instrument was made based on the best current knowledge. After individual searches, some tools were excluded as they were replaced with a newer, improved version.

### Selecting the studies

We imported all titles of our search into Rayyan software and deleted duplicates [51]. Reviewers in pairs (MSo and MBF, AS and MSe) read the titles and abstracts of the studies found following the search strategy to determine their eligibility. Then, studies were categorized as “include” or “exclude.” In the event of contradictory information or disagreement, all the authors responsible for the publication made a final decision after a discussion. Finally, full texts of the selected studies were retrieved for a final review and distributed among the researchers in the same pairs. As before, authors jointly decided to include or exclude given publication for this scoping review in case of doubt.

### Charting the data

Data from all studies included in the review were extracted and collected in an Excel spreadsheet to create an appropriate profile for each tool and determine its suitability for use in a primary care setting. The spreadsheet presents information about the purpose of the instrument, children age range, required time to complete the questionnaire, information whether an assessment report (e.g., filled in by a parent or guardian) or a direct assessment was used (e.g., observation of a child’s behavior), and its psychometric and diagnostic properties. We were also interested in knowing whether any cultural changes were made in a given questionnaire adaptation. The same pairs of reviewers involved in the study selection extracted data from selected studies using an Excel sheet and discussed the discrepancies. To calibrate our data extraction, MSo prepared a calibration exercise on five studies, which improved data extraction.

### Collating, summarizing and reporting the results

After extracting the data, we created tool profiles to standardize the available information about their characteristics, properties, and application in primary care. Each tool that met the inclusion criteria for the study received its profile with data on the name, abbreviation, time of completing the questionnaire, and the person responsible for completing it. In addition, each adaptation of the questionnaire received its line on the spreadsheet for the country for which the validation was prepared, the language into which the text was translated, psychometric and diagnostic data (i.e., reliability, sensitivity, specificity, positive and negative predictive value), and the population in which the study was conducted (with an indication of the specific features of this population). Additionally, we marked in the spreadsheet whether a given version of the questionnaire is the original version and whether the adaptations were subject to linguistic and cultural changes. Figures were rounded to the second decimal point.

## Results

**Fig. 1:**
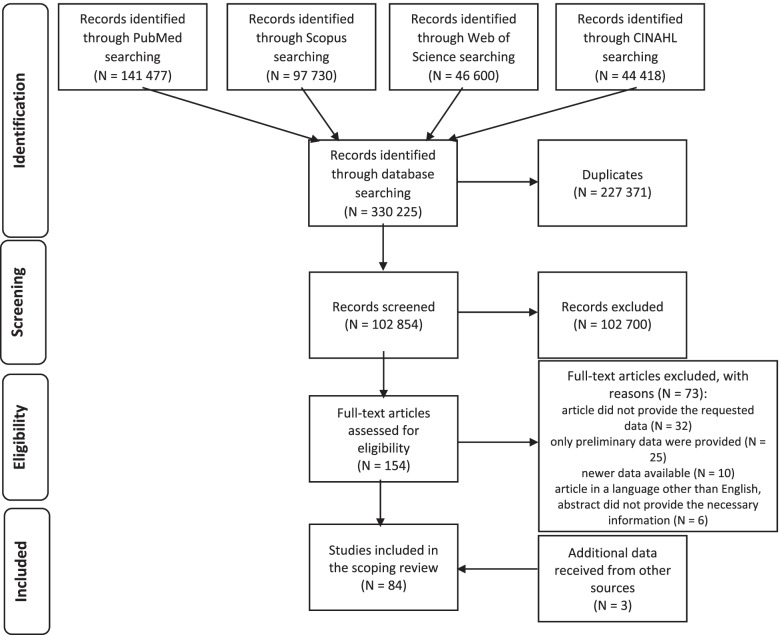
Prisma flow diagram

The initial search yielded 330,225 titles, of which 227,371 were duplicates. After the first screening of titles and abstracts, we assessed 154 full text studies and finally identified 81 studies, which met inclusion criteria and underwent full data extraction. Three additional data sources were included outside of database searches, e.g. test manuals available on-line (see Fig. 1). All collected data are presented in Table 1.

**Table 1:** Overview of available tools for the screening of autism spectrum disorders that met the study criteria

Full name of the tool	Abbreviation	Administer time (in minutes)	Age of the tested child (in months)	Person completing the questionnaire	Country	Language	Reliability (Cronbach’s alpha)	Sensitivity
23-Item Screener	**23Q**	NDA	24–108	parents	Uganda	Luganda	NDA	0.8
Autism Observation Scale for Infants	**AOSI**	20	6–18	health professionals	Canada	English	0.68–0.94	0.84
Autism Parent Screen for Infants	**APSI**	10–15	6–24	parents	Canada	English	0.77–0.92	0.67
Baby and Infant Screen for Children with aUtism Traits	**BISCUIT**	30	17–37	parents or caregivers	USA	English	NDA	0.67–0.94
Behavior Development Screening for Toddlers	**BeDevel**	18–42	10–15	parents or primary caregivers	South Korea	Korean	0.87–0.96	0.83
Brief Autism Detection in Early Childhood	**BADEC**	10–25	12–36	observers	Australia	English	NDA	0.81
Brief Infant Toddler Social Emotional Assessment	**BITSEA**	15	12–36	parents	Finland	Finnish	0.75	NDA
					France	French	0.65–0.79	NDA
					Netherlands	Dutch	0.61–0.76	NDA
					Turkey	Turkish	0.72–0.82	0.83–0.90
					USA	English	0.79	0.67–0.95
Chandigarh Autism Screening Instrument	**CASI**	15–20	18–120	parents	India	Hindi	0.86	0.89
Checklist for Autism Spectrum Disorders	**CASD**	15	12–204	observers	USA	English, Spanish	0.97	0.86
Checklist for Early Signs of Developmental Disorders	**CESDD**	NDA	3–39	observers	Belgium	Dutch	NDA	0.80
Communication and Symbolic Behavior Scale-Infant and Toddlers Checklist	**CSBS-DP**	5–10	6–24	parents	Australia	English	0.82	NDA
					South Korea	Korean	0.90	NDA
					Taiwan	Chinese	0.77	NDA
					USA	English	0.87	0.89
Developmental Behavior Checklist‐Early Screen	**DBC-ES**	5–10	18–48	parents or teachers	Australia	English	0.87	0.75
Early Screening Autistic Traits Questionnaire	**ESAT**	10	14–15	parents	Netherlands	Dutch	NDA	0.68
					Norway	Norwegian	NDA	NDA
First Year Inventory	**FYI**	30	12	parents	China	Chinese	The study was aimed at determining the cut-off points for the study population	
					Israel	Hebrew	0.69	The study was aimed at determining the cut-off points for the study population
					Italy	Italian	The study was designed to test the stability of the cross-cultural measurement between American and Italian children	
					USA	English	0.81	0.44 (0.41 in sample of high-risk children)
					USA	Spanish	The study aimed to identify possible difficulties related to translating the ASD screening questionnaires to adapt them to other languages and cultures	
INCLEN Diagnostic Tool for Autism Spectrum Disorder	**INCLEN-ASD**	45–60	24–108	observers	India	English, Gujarati, Hindi, Khasi, Konkani, Malayalam, Odia, Telugu, Urdu	0.96	0.98
Joint attention-observation schedule	**JA-OBS**	5–10	30	nurses	Sweden	English, Swedish	0.93	0.86
Modified-Checklist for Autism in Toddlers (Revised)	**M-CHAT (R/F)**	5–10	16–30	parents	Albania	Albanian	0.737	NDA
					Argentina	Spanish	0.76	NDA
					Brazil	Portugese	0.95	0.94
					Chile	Spanish	0.89	1.0
					China	Chinese (Mandarin)	0.57	0.96
					Egypt	Arabic	Version of the questionnaire during the assessment of psychometric values—only preliminary results are presented	
					Egypt, Jordan, Kuwait, Lebanon, Oman, Qatar, Saudi Arabia, Syria, Tunisia,	Arabic	NDA	0.87–0.95
					France	French	NDA	0.67
					Iceland	Icelandic	NDA	0.62
					Indonesia	Indonesian	NDA	0.89
					Iran	Kurdish, Persian	0.61	0.90
					Israel	Hebrew	NDA	0.70
					Japan	Japanese	0.56	0.72–0.77
					Malaysia	Chinese, English, Malay	NDA	0.18–0.64
					Mexico	Spanish	0.76	NDA
					Nepal (Bhutan/USA)	Nepali	The study aimed to demonstrate the need for further research on the cultural and linguistic adaptation of the screening questionnaires and the simplification of the wording used in	
					Netherlands	Dutch	NDA	NDA
					Norway	Norwegian	NDA	0.34
					Serbia	Serbian	0.91	NDA
					Spain	Spanish	0.59 (without FUI), 0.62 (with FUI)	0.79
					South Korea	Korean	Only item factor analysis was made	NDA
					Sri Lanka	Sinhala	NDA	0.25
					Sweden	Swedish	NDA	0.77
					Taiwan	Chinese	0.8	0.77–0.88
					Thailand	Thai	NDA	0.91
					Turkey	Turkish	0.67	1.0
					USA	English	0.85	0.91
					Vietnam	Vietnamese	NDA	NDA
Pictorial Autism Assessment Schedule	**PAAS**	15–20	18–40	parents	Sri Lanka	Sinhala, Tamil	0.96	0.89
Quantitative Checklist for Autism in Toddlers	**Q-CHAT**	5	18–24	parents	Iran	Persian	0.89	0.96
					Italy	Italian	0.68	NDA
					Serbia	Serbian	> 0.81	0.96
					Singapore	English	0.53–0.60	NDA
					South Korea	Korean	0.66	NDA
					United Kingdom	English	0.67–0.83	0.44
Quantitative Checklist for Autism in Toddlers – 10-items	**Q-CHAT-10**	< 5	18–24	parents	Chile	Spanish	0.85	0.93
					Serbia	Serbian	> 0.81	0.39
					United Kingdom	English	0.88	0.91
Rapid Interactive Screening Test for Autism in Toddlers	**RITA-T**	10	18–36	doctors	Lebanon	Arabic	0.91	0.96
					USA	English	NDA	1.00
Social Attention and Communication Study	**SACS**	5	12–24	nurses and other health professionals	Australia	English	0.88	0.84
					China	Chinese	NDA	0.53
Screen for Social Interaction	**SSI**	15	24–61	parents or caregivers	USA	English	0.756	0.58–0.94; 0.87–0.81
Screening Tool for Autism in Two-Year-Olds	**STAT** **(T-STAT in Taiwan)**	20	24 (12–24) (18–24 in T-STAT)	examiner	Taiwan	Chinese	0.90	0.93
					USA	English	1.00	1.00 (0.95 for children < 24 months)
Three‐Item Direct Observation Screen	**TIDOS**	15–20	18–60	trained pediatric-oriented professionals	Turkey	Turkish	NDA	0.8
Young autism and other developmental disorders checkup tool	**YACHT-18**	10	18	nurses	Japan	Japanese	NDA	0.80

Full name of the tool	Specificity	Positive predictive value	Negative predictive value	Population and subgroups size	Changes made in the questionnaire			References
					**Original version**	**Lingual changes**	**Cultural changes**	
23-Item Screener	0.77	0.23	0.98	1169 from general population	+			[52]
Autism Observation Scale for Infants	0.98	NDA	NDA	In first study 92 infants siblings of children with ASD for the assessment of reliability, in second—150 infants siblings of children with ASD	+			[53, 54]
Autism Parent Screen for Infants	0.87	0.43–0.79	0.87–0.99	283 in total (79 low risk, 204 high risk)	+			[55]
Baby and Infant Screen for Children with aUtism Traits	0.74–0.89 depending on cut-offs	NDA	NDA	3062 in total (499 with ASD, 383 with PDD-NOS and 2180 with non-ASD related atypical development)	+			[56]
Behavior Development Screening for Toddlers	0.81	0.80	0.83	155 in total (75 ASD, 55 TD, 25 DD)	+			[57]
Brief Autism Detection in Early Childhood	0.78	0.81	0.78	270 in total (95 with ASD, 28 with PDD-NOS, 69 ODD, 78 TD)	+			[58]
Brief Infant Toddler Social Emotional Assessment	NDA	NDA	NDA	50 from general population		Translated with some language changes		[59, 60]
	NDA	NDA	NDA	589 from general population		Translated according to international guidelines		[61]
	NDA	NDA	NDA	3170 from general population		Translated according to international guidelines		[62]
	0.88–0.91	NDA	NDA	462 from general population		Translated with “minimal corrections”		[63]
	0.68–0.95	0.74–0.93	0.75–0.93	1788 from general population	+			[64]
Chandigarh Autism Screening Instrument	0.89	0.67	0.96	405 in total (75 with intellectual disability, 83 with ASD, 87 with DD and 160 TD)	+			[65]
Checklist for Autism Spectrum Disorders	1.00	1.00	1.00	2469 (1052 with ASD, 925 TD, 55 with typical autism and 437 nonautistic clinical children)	+			[66]
Checklist for Early Signs of Developmental Disorders	0.94	0.7	0.99	6808 from general population	+			[67]
Communication and Symbolic Behavior Scale-Infant and Toddlers Checklist	NDA	NDA	NDA	1725 infants already participating in a longitudinal study of language development				[68]
	NDA	NDA	NDA	219 of TD children				[69]
	NDA	NDA	NDA	171 from general population		Translation was made	Substitution of English phonemes with suitable Chinese phonemes; replacement of frequently used words (for example “uh/oh” was replaced with “thank you”)	[70]
	0.89	0.94	0.80	2454 in the reliability study, 3026 (3021 of children not previously identified, 5 with DD) in the study of diagnostic properties	+			[71–73]
Developmental Behavior Checklist‐Early Screen	0.51	0.77	0.48	207 children with or suspected of DD	+			[74]
Early Screening Autistic Traits Questionnaire	0.96	0.10	0.99	31,724 from general population	+			[75]
	NDA	0.07–0.3	NDA	12,666 from general population		Translation only		[76]
First Year Inventory				518 from general population		Translation only		[77]
				471 from general population		Items were culturally and linguistically adapted		[78]
				657 from general population		Translation only		[79]
	0.99 (0.81 in sample of high-risk children)	0.31 (0.52 in sample of high-risk children)	0.99 (0.73 in sample of high-risk children)	1496 from general population for study of test reliability; 699 from general population for other properties; 121 in the study on high-risk sample	+			[80–82]
				25		Items were culturally and linguistically adapted		[83]
INCLEN Diagnostic Tool for Autism Spectrum Disorder	0.95	0.91	0.99	In first round of the study – 266 in total (81 with ASD, 120 NDDs, 65 TD); in second round – 154 (90 with average and 64 with subnormal intelligence)	+			[84]
Joint attention-observation schedule	NDA	0.93	NDA	3999 from general population	+			[85]
Modified-Checklist for Autism in Toddlers (Revised)	NDA	0.895	NDA	2594 from general population		Translation only		[86]
	NDA	NDA	NDA	420 from general population		New translation was completed to adapt it to the Spanish used in Argentina, making slight changes to idiomatic turns of phrases and different expressions closer to Argentine vocabulary, for example: item “plaza” (square) was changed into “parque”	Throughout the entire questionnaire, the words “hijo/hija” (son/daughter) were replaced by “nino/nina” (boy/girl), so that the questionnaire could be administered in the case of another relative taking the child to a pediatric check-up	[87]
	0.91	0.86	0.97	303 from general population		The older version of the M-CHAT test was used in the study, no data available on cultural changes		[88]
	0.98	NDA	NDA	120 in total (20 with suspected ASD, 100 TD)		Semantic changes in 15 out of 20 items, grammatical changes		[89]
	0.86	0.07	1.0	7928 from general population		Translation in accordance with the principles semantic and linguistic of equivalence	Adapting children's behavior to culture: for example—“vacuum the rug” and “mow lawn” are not common activities in China, so we use “wipe the table” instead	[90]
				5546 from general population		Translation only		[91]
	0.76–0.89	0.82–0.9	0.86–0.93	228 in total (122 with ASD, 106 TD)		Translators added the specific dialect of some words to the classical Arabic to clarify the meaning of a number of items of the questionnaire		[92]
	0.94	0.14	0.99	1250 from general population		Translation only		[93]
	0.99	0.72	0.99	1585 from general population		Translation with minor changes	Minor cultural adaptation in follow-up interview – “an action figure” was specified as a “Lego or Playmobil figure”	[94]
	0.95	0.76	0.98	168 from general population without severe sensory and communication disability or ASD diagnosed before		Translation only		[95]
	0.82	0.05 (without FUI)	NDA	2941 from general population		Translation only		[96]
	0.98	0.20	1.00	1591 from general population		In this study the original version of M-CHAT was used		[97]
	0.84	0.08–0.12	NDA	1851 from general population; in reliability study – 24 children		NDA	Authors added some illustrations in order to encourage caregivers to notice negative symptoms	[98, 99]
	1.00	0.26–0.78	0.99	19,297 from general population		Translation only		[100]
	NDA	NDA	NDA	456 in total (117 high-risk, 339 TD)		Translation with minor cultural adjustments	Describing the “peek-a-boo” game (Mexican parents do not have a specific name for it)	[101]
				13 pediatric Nepali refugee patients living formerly in Bhutan (from general population)		Translation only		[102]
	NDA	0.01–0.1	> 0.98	12,102 from general population		Translation only		[76]
	0.93	0.02	NDA	52,026 from general population		Translation only		[103]
	NDA	NDA	NDA	148 in total (128 from general population, 20 high-risk)		Slight adaptation of wording was required due to languages differences		[104]
	0.99	0.39	0.99	6625 from general population		Translation only		[105]
	NDA	NDA	NDA	2300 from general population		Translation with revision of ten Korean mothers living in Sothern California		[106]
	0.71	0.13	0.85	374 from general population (28 with “red flags”)		Translation only		[107]
	NDA	0.92	NDA	3999 from general population		A few minor adjustments of the Swedish language were done		[85]
	0.53–0.72	0.63–0.72	0.77–0.82	236 of ASD high-risk sample		Translation only		[108]
	1.0	0.96	0.99	841 in total (109 high-risk, 732 low-risk)		To increase the suitability of the M-CHAT in a Thai cultural context, authors hypothesized that a screening process that includes both a parent-completed questionnaire followed by a semi-structured interview by trained clinicians, only for cases that initially screen positive that could improve overall sensitivity, specificity, PPV and NPV		[109]
	0.91	0.09	1.0	6712 from general population		Translation only		[110]
	0.99	0.11 (without follow-up interview (FUI))/ 0.65 (with FUI)	0.99	3793 in total (3309 low-risk, 484 high-risk)	+			[111]
	NDA	0.763	NDA	6583 from general population				[112]
Pictorial Autism Assessment Schedule	0.61	0.78	0.77	105 in total (45 with ASD, 30 DD, 30 TD)	+			[113]
Quantitative Checklist for Autism in Toddlers	0.90	NDA	NDA	100 in total (50 TD, 50 ASD)		Translation only		[114]
	NDA	NDA	NDA	2400 from general population		Translation only		[115]
	0.82	NDA	NDA	220 in total		No access to the full content of the article		[116]
	NDA	NDA	NDA	514 from general population		NDA		[117]
	NDA	NDA	NDA	104 in total (24 ASD, 80 unselected)		Translation only		[118]
	0.98	0.28	NDA	In first study – 795 in total (754 from unselected group, 41 ASD); in second study 3770 from general population	+			[119, 120]
Quantitative Checklist for Autism in Toddlers – 10-items	0.77	0.48	NDA	287 in total (125 TD, 149 DD, 13 ASD)		Translation only		[121]
	0.99	1.00	1.00	220 total		No access to the full content of the article		[116]
	0.89	0.58	NDA	880 in total (754 control, 162 ASD)	+			[122]
Rapid Interactive Screening Test for Autism in Toddlers	1.00	1.00	0.96	48 in total (19 TD, 29 high-risk)		Translation only		[123]
	0.84	0.88	NDA	61 in total (23 ASD, 19 DD/non-ASD, 19 TD)	+			[124, 125]
Social Attention and Communication Study	0.99	NDA	NDA	First study—20,770 from general population, second study – 99 identified as “at risk” in first study	+			[126, 127]
	1.00	0.42	1.00	10,514 from general population		Translated and evaluated with English version to be comparable in meaning		[128]
Screen for Social Interaction	0.61–0.87; 0.70–0.71	NDA	NDA	In first study – 111 in total (51 high-risk and 60 low-risk), in second study – 350 (168 from general population, 182 high-risk)	+			[129, 130]
Screening Tool for Autism in Two-Year-Olds	0.74	0.94	0.97	107 in total; in first stage—15 with ASD and 15 with DD or LI; in second – 77 with ASD, PDD-NOS or DD/LI		Two items of the questionnaire were changed	The toy that is shown to the child has been changed (from a dog to an elephant)	[131]
	0.85 (0.73 for children < 24 months)	0.86 (0.56 for children < 24 months)	0.92 (0.97 for children < 24 months)	In first research -104 in total (50 with ASD, 15 with PDD-NOS and 39 with DD/LI). In second research – 77 with older sibling with ASD or had been referred for evaluation for concerns about ASD	+			[132, 133]
Three‐Item Direct Observation Screen	0.74	0.6	0.87	259 in total (86 with ASD, 76 with DD without ASD, 97 with typical development)	+			[134]
Young autism and other developmental disorders checkup tool	0.863	NDA	NDA	2814 from general population, without any recognized disease or disorder	+			[135]

*DD* Development delay, *LI* Language impairment, *NDA* No data available, *NDDs* Other neuro-developmental disorders, *PDD-NOS* Pervasive developmental disorder not otherwise specified, *TD* Typical development

### Study characteristics

The studies described research from 37 countries; most studies originated from the US (*N* = 18), Australia (*N* = 5), and South Korea (*N* = 4). In addition, one article reported a study conducted in nine Arabic countries (Egypt, Kuwait, Jordan, Oman, Qatar, Saudi Arabia, Syria, Tunisia, and Lebanon), and one from the US conducted on a group of Nepalese refugees from Bhutan [92, 102]. The number of scientific papers published during the period under review was relatively stable, with an increase over the last five years (2016–2021).

### Study objectives

The studies included in the review had varied purposes; however, a significant majority focused on determining the psychometric values of the tools. Reliability (defined as Cronbach’s alpha) was provided in 46 of all studies (one study reported only the factor analysis of the instrument), sensitivity was assessed in 53 studies, specificity in 51 studies, positive predictive value (PPV) in 47, and negative predictive value (NPV) in 36 studies. Two studies aimed to determine the cut-off points for the study population for a given tool [77, 78]. One study aimed to demonstrate the need for further research on the cultural and linguistic adaptation of screening questionnaires and simplifying the wording used in them [102]. Finally, one study was designed to test the stability of the cross-cultural measurement, and one aimed to identify possible difficulties related to translating the ASD screening questionnaires to adapt them to other languages and cultures [79, 83].

### Study populations

The number of participants included in the studies differed significantly, ranging from 13 to 52,026 [102, 103]. 34 studies included more than 1,000 children, while six had more than 10,000 participants.

Children from the general population were included in 46 studies. In eight papers, the research was based only on a group of children at risk. One study was conducted in a group of typically developing children [69]. In the case of three publications, the characteristics of the studied population were not specified. The remaining publications concerned both children with a low and high risk of ASD. It is worth noting the different understanding of the term “high-risk children” in individual papers, as risk groups, for example, included siblings of children diagnosed with ASD, children already diagnosed with ASD or other developmental disorders, or suspected of developmental delay, etc.

### Tools characteristics

In the course of the study, we were able to identify 26 different autism spectrum disorder screening tools that met our study criteria.

We would like to point out that while researching the information about tools, we found mixed data on the availability of the Checklist for Autism Spectrum Disorder (CASD) for professionals who are not psychologists or have not completed the appropriate training. Nevertheless, we decided to include CASD in this publication as a tool available to PCPs.

### Original versions of questionnaires

The original versions of questionnaires come from 13 countries. Most of them (as much as 35%, *N* = 9) were created in the US. Only two questionnaires were developed in low- and low-middle-income countries (Uganda and Sri Lanka) [52, 113, 136]. An even greater disproportion could be observed in the languages in which the original versions of the tools are available. Of the 35 original language versions (some questionnaires such as CASD, JA-OBS, and PAAS were prepared in two languages, and INCLEN-ASD even in nine), almost half (*N* = 17) were in English.

### Number of language versions and cultural adaptations of ASD screening tools

Data from selected publications allowed us to create 75 profiles of different versions of the adaptations or original versions of ASD screening questionnaires. Most tools were prepared in one country in one language version. At least one questionnaire was tested in a total of 45 different countries. The largest number of various questionnaires was available in the US (11), Australia and South Korea (4 each), China, the Netherlands, and Turkey (3 each).

Some questionnaires in one study were translated into multiple languages simultaneously; however, at least one tool was available in 35 different languages. In some countries, the questionnaires were adapted to the local dialect (e.g., the Spanish versions of M-CHAT were adapted to Spanish, Mexican, Chilean, and Argentinian respondents) [87, 89, 101, 105]. Most of the questionnaires were available in English (*N* = 21), Spanish (*N* = 7), Chinese (*N* = 6), Dutch (*N* = 4) and Korean (*N* = 4).

At this point, it is worth mentioning that there are many translations of the questionnaires, such as M-CHAT or Q-CHAT, available on the websites of organizations involved in developing them. For example, the most popular M-CHAT is available in 73 versions, but most lack research published in international journals [137, 138]. The situation is similar with the Japanese and Spanish BITSEA versions [139]**.**

Most language versions of the individual questionnaires were translated directly into the language of the surveyed population, sometimes with minor changes. However, for example, in the Argentinian version of the M-CHAT questionnaire, the dialect was changed to match better Spanish used in Argentina. Likewise, in the Taiwanese version of STAT, two items were changed to suit the Taiwanese population better [87, 131].

In addition, cultural changes were made in nine adaptations. For example, phonemes were adapted to the language, and the type of assessed play or the type of toy shown to children was changed to capture their interest.

### Psychometric values

When searching for information on different versions of questionnaires, we focused primarily on reliability, sensitivity, specificity, PPV, and NPV. We made the decision not to include validity data in our review due to the considerable variation in the methodology used across studies (different types of validity measured by various means) or other psychometric values (such as positive or negative likelihood ratio) due to the small number of studies containing these data and the desire to simplify the table as much as possible to facilitate its use by practitioners.

Out of all 75 profiles, we were only able to complete 20 of them containing all the five values sought.

#### Reliability

Internal reliability of the test is a measure defining the consistency of items included in a given scale, i.e., it determines to what extent the items included in a given factor or scale are similar to each other or whether they test the same phenomenon. The most common measure of reliability is Cronbach’s alpha (α) [140]. In the profiles we created, this measure ranges from 0.53 to 1.00. Using the rule of thumb and other different qualitative descriptors methods, 6 of the studies had excellent reliability (α > 0.93), 2 – strong (0.91–0.93), 12 – reliable (0.84–0.90), 14 – relatively high (0.70–0.83), and 13 had reliability below 0.70 [141].

#### Sensitivity

Test sensitivity is the ratio of the true positives to the sum of the true positives and the false negatives. A sensitivity of 100% would mean that all individuals with existing disorders would be diagnosed. Values of reported sensitivity in 53 profiles varied from 0.18 to 1.00. Most of the tests (*N* = 42) scored above 0.70. There is a significant discrepancy between the sensitivity values between linguistic adaptations of the same type of questionnaire (e.g., M-CHAT used in the US and Sri Lanka), resulting potentially from an inadequate cultural adaptation of the tool [107, 111].

#### Specificity

Test specificity is the ratio of the true negatives to the sum of the true negatives and false positives. A specificity of 100% would mean that all healthy individuals in the test performed would be marked as healthy. Specificity was calculated for 51 of the above-mentioned versions of questionnaires and ranged from 0.51 to 1.00. In 37, specificity exceeded 0.80.

#### Positive predicting value (PPV)

PPV is equal to the proportion of true positives out of all positives and determines the probability that a positive test result is accurate. PPV of the questionnaires in the studies included in the review ranged from 0.01 to 1.00, showing a significant variety. Noteworthy is the considerable increase in PPV after the follow-up interview was used in the American version of M-CHAT, showing an increase from 0.11 to 0.65 [111].

#### Negative predicting value (NPV)

NPV is the proportion of true negatives out of all negatives; it determines the probability that a negative test result is accurate. All versions of questionnaires, except one (DBC-ES with NPV = 0.48), for which NPV was calculated, had NPV greater than 0.73 [74].

### Person completing the questionnaire

ASD screening questionnaires can generally be divided into questionnaires filled in by people who have constant contact with the child (parents or guardians) or independent observers – specialists (e.g., doctors, nurses, psychologists, etc.). Most (15 out of 26) tools were intended to be filled by parents, and specialists only dealt with possible doubts arising while filling in the questionnaire and calculated the result of the test. These also tools underwent cultural adaptation much more often than those in which a specialist assessed the child. Some instruments were by definition predisposed to a given professional group, e.g., the assessment of a child’s development using the JA-OBS test is performed by nurses [85].

### Time of completing the questionnaire

Most of the questionnaires listed above should not take more than 10–20 min for parents or specialists to complete, and some only take 5 min. For example, according to the authors, the shortened version of Q-CHAT (Q-CHAT-10) takes less time than 5 min [122]. On the other hand, BeDevel can take over 40 min to complete, and INCLEN-ASD takes 45–60 min [57, 84].

## Discussion

Our research revealed many tools for early ASD screening that can be employed in primary care (26 different instruments in 75 adaptations). An ideal tool for ASD screening seems to be a free and short instrument with items suitable for assessing development, with good psychometric properties, corresponding to the entire studied population, using plain language (low-reading level), easy to assess by people with no experience in psychometrics (easily score-able), providing simple and clear guidance on what to do after screening [142]. Unfortunately, it is unclear which existing tools are best suited for this, so further development of both instruments and research into their use is necessary. Furthermore, there is a possibility that it will be appropriate to create an entirely new tool, which will be much more effective than the existing ones. The problem is further exacerbated by the small number of meta-analyses and systematic reports on the effectiveness of given screening tools [143–145]. Compared to previous studies, we were able to collect data on a much larger number of ASD screening tools available, however our results confirm the previous findings that screening tools for ASD are adequate to detect autism at the early stages of life. The APSI, BITSEA, CESDD, CSBS-DP, M-CHAT, SACS, and STAT deserve recognition, as the studies examining these tools had large sample sizes, and they found these tools in particular to have high psychometric and diagnostic values. For this reason, it seems that these mentioned tools can be used in the population-based ASD screening. The new questionnaires (e.g. BeDevel) look promising as effective tools, but more research is required. Furthermore, among all included tools CSBS-DP, M-CHAT, and STAT are recommended by the Centers for Disease Control and Prevention (CDC) for ASD screening in the United States [146]. An additional positive in favor of M-CHAT is the multitude of language versions that were at least partially validated; the second such questionnaire is Q-CHAT. Another issue is that evaluating the usefulness of some of the questionnaires mentioned above is based on studies conducted a long time ago.

From the perspective of primary care workers, it is also important to reduce the occupational encumbrance of implementing another examination tool which is the responsibility of the PCPs. Hence, it seems that it would be favorable for PCPs to implement screening questionnaires filled out by a parent. On the other hand, questionnaires in which a neutral observer assesses the child are slightly more effective in detecting early symptoms of autism spectrum disorders [147].

Still, the main problem for PCPs will be choosing the right tool to carry out ASD screening. Positive experiences from the United States, where a mass ASD screening system was implemented successfully, indicate the suitability of using ASD screening tests in primary health care [40, 44]. Unfortunately, experiences from the US cannot be transferred directly to other countries. Furthermore, it is crucial for early diagnosis of ASD to have tools that respond to cultural and linguistic differences (as well as the local perception of “disability”). Hence, the use of mismatched tools may be inappropriate [46]. For example, in Jamaica, the percentage of parents reporting that their child shows developmental delays compared to peers is significantly higher than in Bangladesh or Pakistan [148]. The global application of ASD screening, especially in low- and middle-income countries (LMICs), is associated with many problems because most existing tools were developed in North America or Europe, but they are used – often without any significant modifications – in countries whose cultures differ significantly from those in which they were created. In particular, our study shows that there is a lack of tools to identify children with ASD in Africa and other LMICs [149, 150]. There are many possible causes of this state—the cultural maladjustment of the existing tools developed in Europe and North America, the lack of funds for research, a smaller number of psychiatrists and psychologists per capita than in Western countries, or less interest in the subject of ASD [150, 151]. The inability to diagnose ASD in LMICs leads to significant burden on quality of life and costs of medical care and special education that these communities are increasingly witnessing. Therefore, further steps (i.e. developing new culturally appropriate tools, increasing research funding) are needed to raise awareness of the early detection of ASD among the LMICs communities.

Another difficulty is the availability of some of the tools. Many of the instruments that met the study criteria are only available for scientific use. And even if access to them is free, it requires contact with researchers and the authors’ consent for further use.

The lack of available screening tests for individual populations, incomplete validation, or limited availability is not the only difficulty in popularizing early diagnosis of ASD. Screening tests have limited sensitivity—some of the children who received negative screening will receive in subsequent years of their life diagnosis of ASD [152]. Hence, it is not only necessary to pay attention to the dissemination of screening but also to remember the necessity of further continuous monitoring (follow-up) of children’s development [152]. The situation is further exacerbated by the fact that there are no readily available (e.g., in the public domain) rapid tests for older children (aged 30–60 months) as is the case with other psychiatric disorders, e.g., Vanderbilt ADHD Assessment Scales for attention-deficit hyperactivity disorder or Screen for Child Anxiety Related Disorders (SCARED) for anxiety disorders. This makes it necessary to decide whether the child should be referred for further tests based solely on the experience of the primary care worker, which may delay the diagnostic process. These limitations can have long-term adverse consequences (e.g. limited availability of screening tools to individual populations, their incomplete validation or the lack of existing guidelines on developmental disorders for GPs) that can lead to a delay in the diagnostic process of ASD, which can significantly increase the age of diagnosis.

This is not the only reason for delayed ASD diagnosis in children. The example of the United States demonstrates that causes for delay may be due to imperfections of the public health system and low predictive values of the tests (especially in children scoring close to the cut-off limits). Examples of such restrictions include the following:

1. not all children receive healthcare as infants,
2. not all children who are receiving healthcare are screened—only 8–28% of pediatricians in the United States use ASD screening tools in their daily practice [153, 154],
3. not all screened children undergo additional consultations in case of a positive result [42, 43] – only 31% of children with a positive screening test were referred for further diagnosis, 20% to an early intervention center and 36% to an audiologist [155]; these values are slightly higher in another study [42].

The data above show that even with the widespread of the idea of ASD screening, it may not be enough for a complete diagnosis of all affected children. In this case, the delay or lack of diagnosis is primarily due to omissions of the diagnostic process on the part of health care workers.

From an ethical point of view, it should be noted that lowering the age at which the diagnostic process begins in the population will result in an increased number of “false positive” cases, which entails a lot of stress experienced by the families of children that could be difficult to counteract in primary health care [33, 49]. Another problem is that it implies the rising cost of additional evaluation processes in children developing correctly.

The most controversial issue regarding universal ASD screening in children is the cost-effectiveness of ASD screening, primarily due to the moderate accuracy of current tools and the low prevalence of the disorder [45] Attempts to estimate the cost-effectiveness of ASD screening indicate that universal screening may not be financially sound mainly due to delays in further diagnostic and therapeutic steps [33]. Eliminating the waiting time for further consultations with simulation models showed that the initial high cost incurred for screening might be offset by future savings resulting from improved functioning of ASD patients in society. However, the same analyses conducted in high-risk children showed the cost-effectiveness of screening. Nevertheless, the significant benefits of early intervention justify attempts to further refine this strategy for the early detection of autism spectrum disorders [156].

### Limitations of the study

There are several limitations to this review. This study includes only scientific publications whose full text was in English or had an abstract containing most of the necessary data to create a profile for the tool. Because the goal of researchers studying early diagnosis of ASD is the implementation of the instruments in a given country, some existing research may have been excluded due to the publication of results of the validation process in languages other than English in local peer-reviewed journals.

The review was carried out mainly by using search string for publications in four scientific databases, potentially limiting the results. We searched for publications over a broad period of time (1980–2021), which increased the number of available manuscripts. This may be a drawback of the research, because we could include in the study tools, the use of which in practice may prove difficult or ineffective.

It should also be noted that researchers carried out the measurements of psychometric and diagnostic properties of various tests in different ways, making it impossible to compare their parameters without taking into account the methodological details contained in the source texts.

During the review process of the article, research on a new, promising screening tool—Early Screening for Autism and Communication Disorders (ESAC) was published. ESAC consists of 46 items, covers children between 12–36 months and has a reliability ranged from 0.92 to 0.95, sensitivity between 0.86 and 0.92 and specificity between 0.74 and 0.85 in an American population [157].

## Conclusions

The results of our review show that there are several diagnostic tools for early ASD screening that can be used in a primary care setting for which the full validation process was carried out and showed high psychometric and diagnostic values. These tools could effectively accelerate the diagnostic process and lead to a faster start of personalized therapy. As some examples show (e.g. Icelandic version of M-CHAT or Taiwanese version of STAT), they could also become the basis for preparing almost equally effective adaptations of screening tests for different populations, especially after introducing cultural and linguistic modifications [94, 131].

Unfortunately, for a large part of the tools, no changes other than accurate translation were made to fit the questionnaire to the characteristics of the particular population. Furthermore, only partial validation studies were carried out in many cases, which means that using them in everyday practice may be ineffective. Finally, the more culturally different two populations are, the more a tool designed for one will be less effective for the other.

Therefore, it appears necessary to continue research on adaptations of existing ASD screening methods and attempt to improve them and constantly increase the knowledge of health care professionals about ASD, improve the follow-up process, and further evaluate the cost-effectiveness of the ASD screening process.

### Practical implications

This review highlights the available options for early diagnosis of ASD in primary care from a global perspective, indicating the importance of psychometric and diagnostic values in choosing the most suitable tool for everyday practice.

## Supplementary Information

**Additional file 1. **

**Additional file 2. **

## Data Availability

All data generated or analysed during this study are included in this published article and its supplementary information files (S1 File).

## Abbreviations

23Q: 23-Item Screener
AAP: American Academy of Pediatric
ADHD: Attention-deficit hyperactivity disorder
AOSI: Autism Observation Scale for Infants
APSI: Autism Parent Screen for Infants
ASD: Autism spectrum disorder
BADEC: Brief Autism Detection in Early Childhood
BeDevel: Behavior Development Screening for Toddlers
BISCUIT: Baby and Infant Screen for Children with aUtism Traits
BITSEA: Brief Infant Toddler Social Emotional Assessment
CASD: Checklist for Autism Spectrum Disorder
CASI: Chandigarh Autism Screening Instrument
CESDD: Checklist for Early Signs of Developmental Disorders
CSBS-DP: Communication and Symbolic Behavior Scale-Infant and Toddlers Checklist
DBC-ES: Developmental Behavior Checklist‐Early Screen
DD: Development delay
ESAC: Early Screening for Autism and Communication Disorders
ESAT: Early Screening Autistic Traits Questionnaire
FYI: First Year Inventory
GP: General Practitioner
INCLEN-ASD: INCLEN Diagnostic Tool for Autism Spectrum Disorder
JA-OBS: Joint attention-observation schedule
LI: language impairment
LMICs: Low and middle income countries
M-CHAT (R/F): : Modified-Checklist for Autism in Toddlers (Revised)
NDA: No data available
NDDs: Other neuro-developmental disorders
NPV: Negative predicting value
PAAS: Pictorial Autism Assessment Schedule
PCP: Primary care physician
PDD-NOS: Pervasive developmental disorder not otherwise specified
PPV: Positive predicting value
Q-CHAT: Quantitative Checklist for Autism in Toddlers
Q-CHAT-10: Quantitative Checklist for Autism in Toddlers – 10-items
RITA-T: Rapid Interactive Screening Test for Autism in Toddlers
SACS: Social Attention and Communication Study
SSI: Screen for Social Interaction
STAT: Screening Tool for Autism in Two-Year-Olds
TD: Typical development
TIDOS: Three‐Item Direct Observation Screen
WHO: World Health Organization
YACHT-18: Young autism and other developmental disorders checkup tool
