# Supplementary material for: Tools for early screening of autism spectrum disorders in primary health care – a scoping review
Source: BMC Prim Care. 2022 Mar 15;23:46. doi: 10.1186/s12875-022-01645-7 (PMC8925080; doi:10.1186/s12875-022-01645-7)
Supplement: Supplementary file 2 — Additional file 2. [file 12875_2022_1645_MOESM2_ESM.docx]

**Supporting information**

**S1 File. Search strings used in the study**.

**PubMed and CINAHL**
"23-Item Screener" OR "Autism Observation Scale for Infants" OR "AOSI" "Autism Parent Screen for Infants" OR "APSI" OR "Baby and Infant Screen for Children with aUtism Traits" OR "BISCUIT" OR "Behavior Development Screening for Toddlers" OR "BeDevel" OR "Brief Autism Detection in Early Childhood" OR "BADEC" OR "Brief Infant Toddler Social Emotional Assessment" OR "BITSEA" OR "Chandigarh Autism Screening Instrument" OR "CASI" OR "Checklist for Autism Spectrum Disorders" OR "CASD" OR "Checklist for Early Signs of Developmental Disorders" OR "CESDD" OR "Communication and Symbolic Behavior Scale-Infant and Toddlers Checklist" OR "CSBS-DP" OR "Developmental Behavior Checklist‐Early Screen" OR "DBC-ES" OR "Early Screening Autistic Traits Questionnaire" OR "ESAT" OR "First Year Inventory" OR "FYI" OR "INCLEN Diagnostic Tool for Autism Spectrum Disorder" OR "INCLEN" OR "Joint Attention-Observation Schedule" OR "JA-OBS" OR "Modified-Checklist for Autism in Toddlers" OR "M-CHAT" OR "Pictorial Autism Assessment Schedule" OR "PAAS" OR "Quantitative Checklist for Autism in Toddlers" OR "Q-CHAT" OR "Rapid Interactive Screening Test for Autism in Toddlers" OR "RITA-T" OR "Social Attention and Communication Study" OR "SACS" OR "Screen for Social Interaction" OR "SSI" OR "Screening Tool for Autism in Two-Year-Olds" OR "STAT" OR "Three‐Item Direct Observation Screen" OR "TIDOS" OR "Young autism and other developmental disorders checkup tool" OR "YACHT-18"

**Scopus**

( TITLE-ABS-KEY ( "23-Item Screener" )  OR  TITLE-ABS-KEY ( "Autism Observation Scale for Infants" )  OR  TITLE-ABS-KEY ( aosi )  OR  TITLE-ABS-KEY ( "Autism Parent Screen for Infants" )  OR  TITLE-ABS-KEY ( apsi )  OR  TITLE-ABS-KEY ( "Baby and Infant Screen for Children with aUtism Traits" )  OR  TITLE-ABS-KEY ( biscuit )  OR  TITLE-ABS-KEY ( "Behavior Development Screening for Toddlers" )  OR  TITLE-ABS-KEY ( bedevel )  OR  TITLE-ABS-KEY ( "Brief Autism Detection in Early Childhood" )  OR  TITLE-ABS-KEY ( badec )  OR  TITLE-ABS-KEY ( "Brief Infant Toddler Social Emotional Assessment" )  OR  TITLE-ABS-KEY ( bitsea )  OR  TITLE-ABS-KEY ( "Chandigarh Autism Screening Instrument" )  OR  TITLE-ABS-KEY ( casi )  OR  TITLE-ABS-KEY ( "Checklist for Autism Spectrum Disorders" )  OR  TITLE-ABS-KEY ( casd )  OR  TITLE-ABS-KEY ( "Checklist for Early Signs of Developmental Disorders" )  OR  TITLE-ABS-KEY ( cesdd )  OR  TITLE-ABS-KEY ( "Communication and Symbolic Behavior Scale" )  OR  TITLE-ABS-KEY ( csbs )  OR  TITLE-ABS-KEY ( "Developmental Behavior Checklist‐Early Screen" )  OR  TITLE-ABS-KEY ( "DBC-ES" )  OR  TITLE-ABS-KEY ( "Early Screening Autistic Traits Questionnaire" )  OR  TITLE-ABS-KEY ( esat )  OR  TITLE-ABS-KEY ( "First Year Inventory" )  OR  TITLE-ABS-KEY ( fyi )  OR  TITLE-ABS-KEY ( inclen )  OR  TITLE-ABS-KEY ( "Joint Attention-Observation Schedule" )  OR  TITLE-ABS-KEY ( "JA-OBS" )  OR  TITLE-ABS-KEY ( "Modified-Checklist for Autism in Toddlers" )  OR  TITLE-ABS-KEY ( "M-CHAT" )  OR  TITLE-ABS-KEY ( "Pictorial Autism Assessment Schedule" )  OR  TITLE-ABS-KEY ( "Quantitative Checklist for Autism in Toddlers" )  OR  TITLE-ABS-KEY ( "Q-CHAT" )  OR  TITLE-ABS-KEY ( "Rapid Interactive Screening Test for Autism in Toddlers" )  OR  TITLE-ABS-KEY ( "RITA-T" )  OR  TITLE-ABS-KEY ( "Social Attention and Communication Study" )  OR  TITLE-ABS-KEY ( sacs )  OR  TITLE-ABS-KEY ( "Screen for Social Interaction" )  OR  TITLE-ABS-KEY ( "Screening Tool for Autism in Two-Year-Olds" )  OR  TITLE-ABS-KEY ( "Three‐Item Direct Observation Screen" )  OR  TITLE-ABS-KEY ( tidos )  OR  TITLE-ABS-KEY ( "Young autism and other developmental disorders checkup tool" )  OR  TITLE-ABS-KEY ( "YACHT-18" ) )

**Web of Science**TS=("23-Item Screener" ) OR TS=("Autism Observation Scale for Infants" ) OR TS=(aosi ) OR TS=("Autism Parent Screen for Infants" ) OR TS=(apsi ) OR TS=("Baby and Infant Screen for Children with aUtism Traits" ) OR TS=(biscuit ) OR TS=("Behavior Development Screening for Toddlers" ) OR TS=(bedevel ) OR TS=("Brief Autism Detection in Early Childhood" ) OR TS=(badec ) OR TS=("Brief Infant Toddler Social Emotional Assessment" ) OR TS=(bitsea ) OR TS=("Chandigarh Autism Screening Instrument" ) OR TS=(casi ) OR TS=("Checklist for Autism Spectrum Disorders" ) OR TS=(casd ) OR TS=("Checklist for Early Signs of Developmental Disorders" ) OR TS=(cesdd ) OR TS=("Communication and Symbolic Behavior Scale" ) OR TS=(csbs ) OR TS=("Developmental Behavior Checklist‐Early Screen" ) OR TS=("DBC-ES" ) OR TS=("Early Screening Autistic Traits Questionnaire" ) OR TS=(esat ) OR TS=("First Year Inventory" ) OR TS=(fyi ) OR TS=(inclen ) OR TS=("Joint Attention-Observation Schedule" ) OR TS=("JA-OBS" ) OR TS=("Modified-Checklist for Autism in Toddlers" ) OR TS=("M-CHAT" ) OR TS=("Pictorial Autism Assessment Schedule" ) OR TS=("Quantitative Checklist for Autism in Toddlers" ) OR TS=("Q-CHAT" ) OR TS=("Rapid Interactive Screening Test for Autism in Toddlers" ) OR TS=("RITA-T" ) OR TS=("Social Attention and Communication Study" ) OR TS=(sacs ) OR TS=("Screen for Social Interaction" ) OR TS=("Screening Tool for Autism in Two-Year-Olds" ) OR TS=("Three‐Item Direct Observation Screen" ) OR TS=(tidos ) OR TS=("Young autism and other developmental disorders checkup tool" ) OR TS=("YACHT-18" ))  *AND* **LANGUAGE:**  (English)  *AND*  **DOCUMENT  TYPES:**  (Article)
